# Complete mitochondrial genome of *Acrossocheilus yunnanensis* (Cypriniformes: Cyprinidae: *Acrossocheilus*) and its phylogenetic analysis

**DOI:** 10.1080/23802359.2020.1780973

**Published:** 2022-10-03

**Authors:** Qiliang Chen, Xiaolong Lian, Yingwen Li, Zhihao Liu, Yanjun Shen

**Affiliations:** Chongqing Key Laboratory of Animal Biology, School of Life Sciences, Chongqing Normal University, Chongqing, China

**Keywords:** *Acrossocheilus yunnanensis*, Cyprinidae, mitochondrial genome, phylogenetic

## Abstract

*Acrossocheilus yunnanensis* is an endemic species in China. In this study, the complete mitochondrial genome of *A. yunnanensis* was determined. It was 16,587 bp in length, containing 13 protein-coding genes, 2 rRNA genes, 22 tRNA genes, and a putative control region. Phylogenetic analysis showed that *A. yunnanensis* was clustered with *A. monticola*.

*Acrossocheilus yunnanensis* is an endemic species in China, which is distributed in the Pearl River system and the upper and middle reaches of the Yangtze River (Li et al. 2019). In recent years, artificial breeding of this fish species has been successful and artificial cultivation has been carried out in some areas of Yunnan Province, China. Mitochondrial genome can provide sufficient resources for genome-wide evolutionary studies and has demonstrated the potential to resolve phylogenetic relationships at different taxonomic levels, and understand the structure and functional evolution (Zhang and Shen 2019; Shen et al. 2020; Yang et al. 2020). In this study, the complete mitochondrial DNA sequence of *A. yunnanensis* and its phylogenetic relationship were determined, which provides useful genetic information for the study on molecular systematics, population genetics, and phylogeography of this species (Ding et al. 2020).

Specimens of *A. yunnanensis* were collected from the Changxi River (108°05′E, 29°06′N), Pengshui, Chongqing, and stored in the Chongqing Normal University Museum (Voucher number: CQN20191109). The complete genome sequence of *A. yunnanensis* was 16,587 bp in length (GenBank: MT476484) and contained 13 protein-coding genes, 2 rRNA genes, 22 tRNA genes, and one displacement loop (D-loop). Most genes were encoded on the H-strand, except the ND6 and eight tRNA genes. Overall base composition of the mitogenome was 31.43% A, 24.59% T, 16.07% G, 27.92% C. The total length of the 13 protein-coding genes was 11,401 bp. Similar to other *Acrossocheilus* species, 12 of them started with an ATG codon, while COX1 started with GTG. Stop codons were variable for all protein-coding genes. Eight genes (ND1, COX1, ATP8, ATP6, COX3, ND4L, ND5 and ND6) used complete stop codon TAA and one gene (ND2) ended with TAG, whereas other four genes (COX2, ND3, ND4 and CYTB) ended with an incomplete stop codon T—, which might be completed by post transcriptional polyadenylation with poly A tail (Ojala et al. 1981).

To confirm the phylogenetic position of *A. yunnanensis* in genus *Acrossocheilus*, a maximum likelihood (ML) phylogenetic analysis was constructed in PhyML 3.0 based on the concatenated dataset of 13 protein-coding genes (PCG) of other 15 *Acrossocheilus* species and two Cyprinidae species (*Cyprinus carpio* and *Carassius auratus*) were used as outgroup. The result showed that the *A. yunnanensis* was well grouped with *A. monticola* (BP = 100) (Figure 1) and the tree topology was congruent with traditional taxonomy.

**Figure 1.**
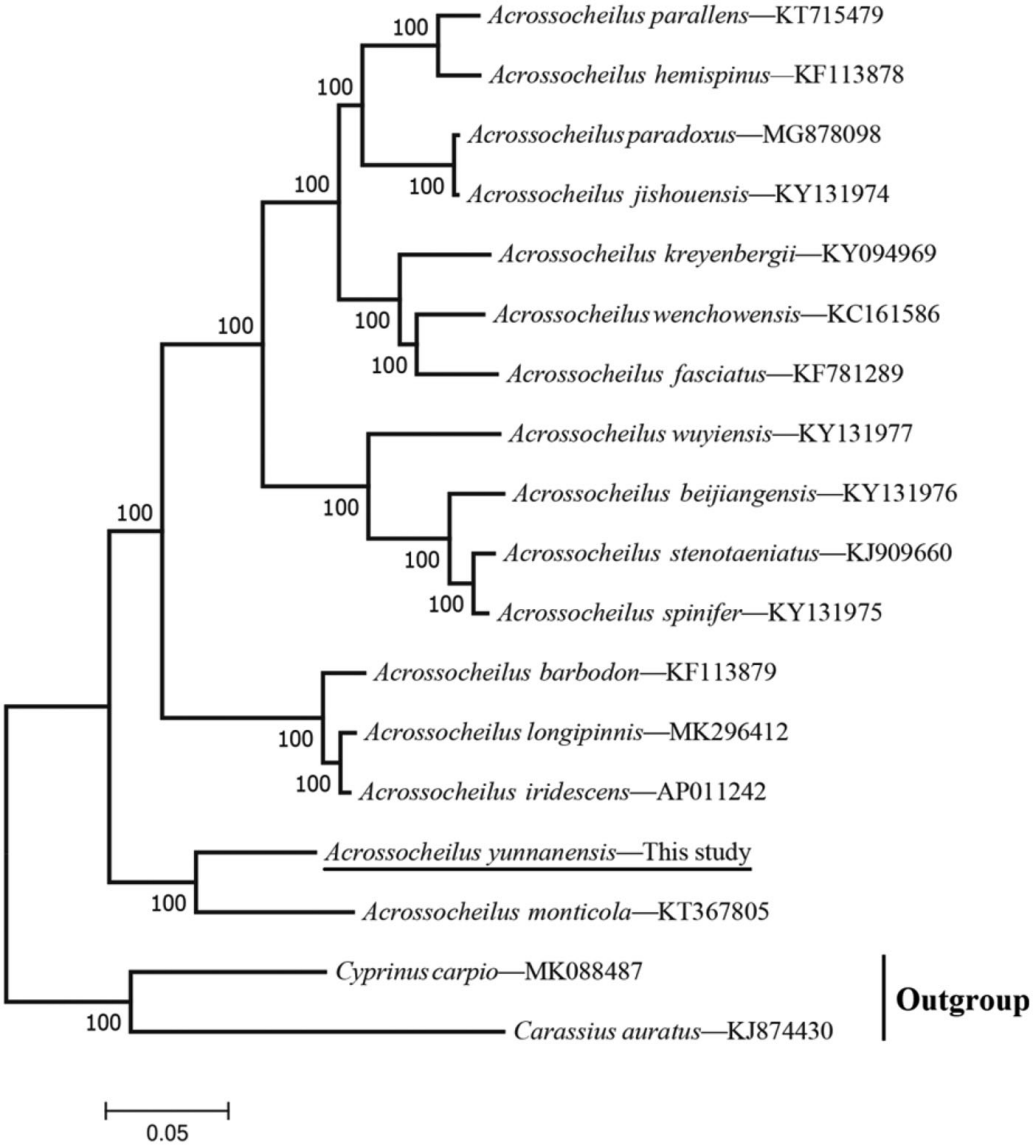
Maximum likelihood (ML) tree showing the phylogenetic position of *Acrossocheilus yunnanensis* among *Acrossocheilus* species based on a dataset of 13 PCGs sequences.

## Data Availability

The data that support the findings of this study are openly available in GenBank of NCBI at https://www.ncbi.nlm.nih.gov, reference number MT476484.

## References

[CIT0001] Ding JH, Yang Y, Li J. 2020. Complete mitochondrial genome of *Iotaphora admirabilis* (Lepidoptera: Geometridae). Mitochondrial DNA Part B. 5(2):1425–1426.

[CIT0003] Li C, Chen K, Qian Z, Zeng B, Song P, Jiang Z. 2019. The complete mitochondrial genome of *Acrossocheilus yunnanensis* (Teleostei: Cypriniformes: Cyprinidae) and its phylogenetic position. Mitochondrial DNA Part B. 4(2):4061–4062. 33366318 10.1080/23802359.2019.1688100 PMC7707677

[CIT0004] Ojala D, Montoya J, Attardi G. 1981. tRNA punctuation model of RNA processing in human mitochondria. Nature. 290(5806):470–474. 7219536 10.1038/290470a0

[CIT0005] Shen YJ, Wang J, Zhang FB. 2020. Complete mitochondrial genome of *Parabotia bimaculata* (Cypriniformes: Cobitidae: Botiinae), an endemic riverine loach in China and phylogenetic analysis for Botiinae. Thalassas 1-7.

[CIT0006] Yang N, Li YW, Liu ZH, Chen QL, Shen YJ. 2020. The complete mitochondrial genome of *Cobitis macrostigma* (Cypriniformes: Cobitidae: Cobitinae) and a phylogenetic implication for its closely related species. Biologia. 75(3):393–399.

[CIT0007] Zhang FB, Shen YJ. 2019. Characterization of the complete mitochondrial genome of *Rhinogobius leavelli* (Perciformes: Gobiidae: Gobionellinae) and its phylogenetic analysis for Gobionellinae. Biologia. 74(5):493–499.

